# Does lipid lowering therapy improve calf muscle perfusion and cellular metabolism in peripheral arterial disease?

**DOI:** 10.1186/1532-429X-11-S1-O61

**Published:** 2009-01-28

**Authors:** Amy M West, Justin D Anderson, Frederick H Epstein, Craig H Meyer, Klaus D Hagspiel, Stuart S Berr, Nancy L Harthun, Arthur L Weltman, Joseph M DiMaria, Jennifer R Hunter, John M Christopher, Christopher M Kramer

**Affiliations:** University of Virginia, Charlottesville, VA USA

**Keywords:** Simvastatin, Peripheral Arterial Disease, Ezetimibe, Tissue Perfusion, Lipid Lowering Therapy

## Introduction

Previous studies suggest that lipid lowering therapy improves symptoms and walking performance in patients with peripheral arterial disease (PAD). We studied the relationship between LDL reduction and both tissue perfusion and cellular metabolism in PAD using magnetic resonance imaging (MRI) and spectroscopy (MRS).

## Methods

61 patients with mild-to-moderate symptomatic PAD (mean age 63 ± 10 years, ankle brachial index 0.69 ± 0.15) were studied before and 1 year after starting one of 3 lipid lowering therapies. Statin-naïve patients were randomized to simvastatin 40 mg or simvastatin 40 mg plus ezetimibe 10 mg (n = 31) and patients already on a statin were given open-label ezetimbibe 10 mg (n = 30). Lipid measurements were obtained as part of the VAP test. Patients with interval stenting of the leg studied (n = 4) or bypass surgery (n = 1) were excluded from analysis. All 56 remaining patients had calf muscle phosphocreatine recovery time constant (PCr) measured using ^31^phosphorus (P) MRS immediately after symptom-limited calf muscle exercise using a MR compatible ergometer on a Siemens Sonata 1.5 T scanner. Exercise time was recorded. ^31^P MRS was obtained using a single-pulse, surface coil localized, 512 ms free induction decay acquisition with 20 averages centered on the mid-calf. PCr was then calculated using a monoexponential fit of phosphocreatine concentration versus time, beginning at cessation of exercise. Calf muscle tissue perfusion (TP) was measured in 50 patients using first-pass contrast-enhanced MRI at peak exercise. The remaining 6 patients were excluded due to compromised renal function. The patients pushed a MR-compatible foot pedal ergometer at a steady rate (10–12 bpm) until limiting symptoms or exhaustion while in a Siemens Avanto 1.5 T scanner and gadolinium (0.1 mM/kg) was infused at peak exercise. Work expended during exercise was recorded. Time intensity curves were generated with ARGUS image analysis software from the region of calf muscle with the greatest intensity post contrast and the slope of this curve was defined as TP. To assess microvascular blood flow within the calf muscle, TP was indexed to macrovascular blood flow into the calf by dividing it by the slope of the popliteal arterial input curve to obtain a perfusion index (PI). Changes in all parameters from baseline to year one were compared by paired t-test and between group differences by unpaired t-test.

## Results

LDL at baseline was 110 ± 34 and was lower at one year (79 ± 34 mg/dl, p < 0.0001). The total cholesterol at baseline was 183 ± 42 and decreased significantly at one year to 146 ± 42 mg/dl, p < 0.0001 as did triglycerides (165 ± 119 to145 ± 81 mg/dl, p = 0.05). There was no change in HDL from baseline to follow-up (44 ± 14 to 43 ± 14 mg/dl, p = NS). See Table 1 for changes in TP, PI, PCr, exercise time and work expended between baseline and year one. A trend was noted towards an increase in exercise time for ^31^P MRS. There were no between group differences in any MR or exercise outcome parameter in patients treated with different lipid lowering regimens.

**Table 1:** Changes in perfusion, metabolism, and exercise parameters over time

	Baseline	Follow up
Perfusion Work, j	225.6 ± 156.1	250.8 ± 293.9
Tissue perfusion	6.45 ± 3.42	6.32 ± 3.44
Perfusion index	0.57 ± 0.39	0.60 ± 0.42
PCr Exercise, sec	156.6 ± 58.8	173.1 ± 57.9*
PCr, sec	85.9 ± 59.3	85.8 ± 59.8

*p = 0.06 vs. baseline

## Conclusion

Lipid lowering therapy over the course of one year in PAD did not improve tissue perfusion as measured by first pass contrast-enhanced MRI or cellular metabolism as measured by phosphocreatine recovery kinetics, although exercise time tended to improve. Thus, the previously demonstrated increase in exercise capacity with LDL lowering in PAD is unlikely due to improvements in tissue perfusion or skeletal muscle metabolism, suggesting that other potential mechanisms of benefit must be at play.

